# Effects of Multiple Treatments of Formic Acid on the Chemical Properties and Structural Features of Bamboo Powder

**DOI:** 10.3390/molecules30020398

**Published:** 2025-01-18

**Authors:** Hui Qiao, Yue Liu, Yongshun Feng, Xin Pan, Xiaowei Zhuang, Jia Ouyang

**Affiliations:** 1 Zhejiang Academy of Forestry, Liuhe Road 399, Hangzhou 310023, China; qiaohui123@njfu.edu.cn (H.Q.); fengyongshun@zjforestry.ac.cn (Y.F.); panxin@zjforestry.ac.cn (X.P.); 2 College of Environmental Science and Engineering, Liaoning Technical University, Zhonghua Road 47, Fuxin 125105, China; 18852677359@163.com; 3 Jiangsu Co-Innovation Center of Efficient Processing and Utilization of Forest Resources, Nanjing Forestry University, Longpan Road 159, Nanjing 210037, China

**Keywords:** formic acid pretreatment, bamboo powder, physicochemical structure, enzymatic hydrolysis

## Abstract

Under mild conditions, formic acid effectively separates the components of lignocellulose, removing the majority of the hemicellulose and lignin from the cellulose. However, it has not yet been determined if multiple treatments with fresh formic acid may totally remove hemicellulose and lignin. In this study, fresh formic acid was used to repeatedly pretreat the bamboo powder, and the effect of multiple treatments on the physicochemical structure of the bamboo powder was investigated using changes in fractions, enzymatic hydrolysis, hydrophilicity, cellulose crystallinity, and lignin structure. Although the hydrophilicity of the powder rose as the number of treatments increased and the number of β-O-4 links in the lignin decreased, it was found that the bamboo powder still contained 5.4% lignin and 2.5% hemicellulose. The 48 h enzymatic yield increased with the number of treatments, with a 59.3% yield obtained at the fifth cycle. This study can serve as a foundation for further research into the mechanism of the influence of organic solvent pretreatment on lignocellulose structural integrity.

## 1. Introduction

Bamboo is a kind of gramineous plant with similar characteristics to woody plants, which are often used as raw materials for paper, food, construction, and daily necessities. According to research reports, bamboo is the fastest growing plant on the earth and can be harvested within 1–5 years [1]. Bamboo resources are widely distributed in China, with an area of 6.73 million hm^2^ per month, accounting for one-third of the world’s bamboo forests. However, the utilization rate of raw bamboo in the bamboo processing industry is only 30–50%, resulting in a large amount of bamboo waste [2]. Bamboo is rich in carbohydrates, and with cellulose and hemicellulose levels accounting for 35–50% and 15–35%, respectively, of the plant [3]. Therefore, it is used to produce fermentable sugars [1], prepare furan platform compounds [4], extract XOS [5], and prepare raw materials for cellulose materials, thereby improving the utilization value of bamboo waste. However, the bamboo cell wall’s complex multilayered structure and chemical composition create a unique anti-depolymerization barrier, making bamboo isolation and utilization more difficult. As a result, one of the most important processes in making good use of bamboo is pretreatment.

It was found that organic solvent pretreatment had a good effect on the separation of cellulose, hemicellulose and lignin. Organic solvent pretreatment has the following advantages: (1) Organic solvent pretreatment is an excellent method for obtaining high-purity cellulose with minimal degradation; (2) Organic solvent pretreatment yields a considerable amount of intact high-quality lignin with stable molecular weight, strong hydrophobicity, and no sulfide; (3) Organic solvent pretreatment outperformed traditional pretreatment methods in terms of hemicellulose fractionation efficiency; (4) Organic solvent pretreatment allows for facile solvent recovery by distillation, and the solvent is reused during the pretreatment [6,7,8]. Formic acid is a biomass-derived organic acid capable of providing H^+^ to hydrolyze hemicellulose into monosaccharides or oligosaccharides. Since the Hildebrand solubility parameter of formic acid is close to that of lignin, formic acid is effective in solubilizing lignin, resulting in the separation of cellulose, hemicellulose, and lignin [9,10]. Previous research has shown that formic acid pretreatment produces good results for the separation of bamboo components [11,12]. Formic acid effectively eliminates lignin by breaking interunit bonds in β-O-4′, β-β and β-5′ at atmospheric pressure [11]. Li et al. [13] found that the removal efficiency and chemical structure of lignin differed at various cycle stages of formic acid treatment. The lignin was continually removed as the reaction time rose, the dissolution of lignin was fast at first and then thereafter sluggish, and the molecular weight of dissolved lignin increased with the extension of time. Previous studies have also found that formic acid pretreatment has a good removal effect on hemicellulose and lignin of different biomass [12]. However, it has not been studied whether multiple formic acid pretreatments can completely remove hemicellulose and lignin. Therefore, the purpose of this study is to perform extensive research on the removal efficiency and physicochemical structure changes in lignin and hemicellulose after several treatments with formic acid.

In this study, the same bamboo powder was pretreated multiple cycles using fresh formic acid to explore the changes in its physicochemical structure. The effects of several formic acid treatments on the changes in crystallinity, hydrophobicity, functional groups, and thermal stability of lignin were investigated by XRD, contact angle, FT-IR spectrometer, and thermogravimetric analyzer. In addition, the effects of physicochemical structural changes on enzymatic hydrolysis were investigated.

## 2. Results and Discussion

### 2.1. Chemical Composition Changes in BP After Formic Acid Pretreatment

**Figure 1:**
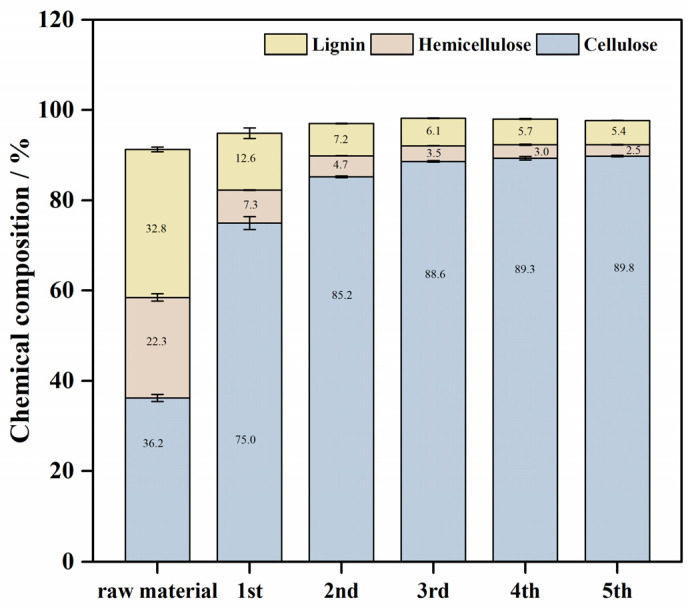
Chemical composition of BPs.

The chemical composition of raw bamboo powder in this study was 36.2% cellulose, 22.3% hemicellulose, and 32.8% lignin. Under mild formic acid treatment conditions, hemicellulose and lignin can be effectively removed. When BP was treated with formic acid for one cycle, 85.0% hemicellulose and 82.5% lignin were removed from the raw material. Correspondingly, the composition of BP was 75.0% cellulose, 7.3% hemicellulose, and 12.6% lignin [14]. In addition, over 96% hemicellulose and 94% lignin were removed after the 5th formic acid pretreatment, and over 80% cellulose was recovered based on the raw material; correspondingly, the fifth batch of BP contained 89.8% cellulose, 2.5% hemicellulose, and 5.4% lignin. As seen in Figure 1, the content of hemicellulose and lignin decreased rapidly after three formic acid pretreatments. After the third pretreatment, the content of hemicellulose and lignin decreased to 3.5% and 6.1%, respectively, indicating that most of the hemicellulose and lignin in bamboo can be dissolved by formic acid. Similarly, it can be seen in Table 1 that the removal of hemicellulose and lignin mainly occurred in the first two pretreatment processes. Compared with the raw materials, the removal rates of hemicellulose and lignin can reach more than 93%. Figure 1 and Table 1 illustrate the chemical composition changes in BP when exposed to multiple cycles of formic acid treatment. In the latter two treatments, only less than 20% of the remaining hemicellulose and lignin were removed (compared to the third sample). From the above results, it appears hemicellulose and lignin in BP cannot be completely removed under mild formic acid pretreatment conditions.

### 2.2. Enzymatic Hydrolysis of Pretreated BPs

**Figure 2:**
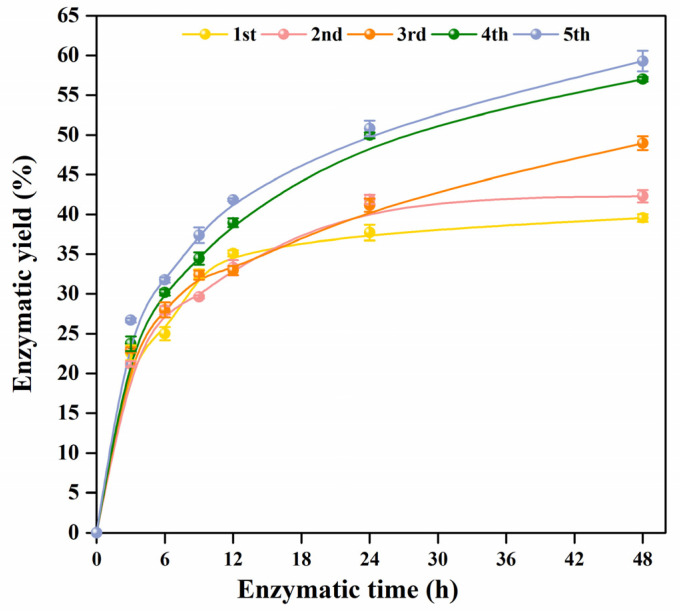
Enzymatic hydrolysis of pretreated BPs within 48 h.

Enzymatic hydrolysis efficiency is one of the characterizations to evaluate the effect of multiple pretreatments. The solid residues obtained by multiple pretreatments were enzymatically hydrolyzed for 48 h, and their enzymatic yields are shown in Figure 2. In the initial 12 h of enzymatic hydrolysis, the five samples were rapidly enzymatically hydrolyzed. After 12 h, the enzymatic hydrolysis rate of 1st and 2nd BP slowed down, while 4th and 5th BP still maintained a faster enzymatic hydrolysis rate. The enzymatic yield increased with the superposition of pretreatment cycles, from 39.6% in the first cycle to 59.3% in the fifth cycle. In addition, with the increase in formic acid treatment cycles, the rate of enzymatic hydrolysis became faster. After the fourth and fifth treatments, the enzymatic hydrolysis yields of 39.0% and 41.8% were obtained at 12 h, which was equivalent to the yield of 1st sample in 48 h enzymatic yield. The above results suggest that as BP was repeatedly treated with formic acid, its hemicellulose and lignin are increasingly stripped from the original structure, and cellulose is also increasingly exposed, thereby increasing the available contact area between cellulose and cellulase, leading to a higher enzymatic hydrolysis yield.

### 2.3. Characterization of Pretreated BPs

**Figure 3:**
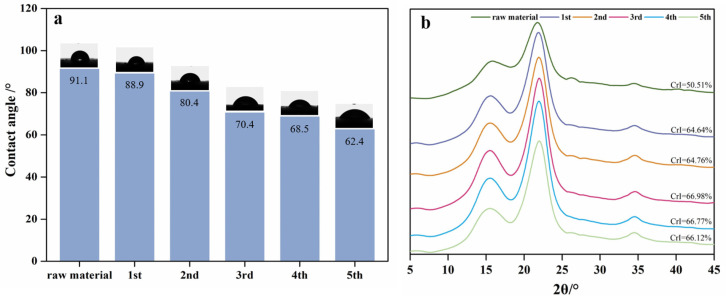
Comparison of pretreated BPs on hydrophilicity and XRD. (**a**) The hydrophilicity of BPs, (**b**) X-ray diffraction (XRD) patterns of BPs.

**Contact Angle** The contact angle was used as a measure of the hydrophilicity of BPs after formic acid pretreatment. The hydrophilic hydroxyl group is easily accessed by water molecules on the BP surface, so that the water droplets diffuse rapidly on the surface, and the contact angle decreases rapidly until the water droplets are completely absorbed [15]. Figure 3a shows the state of water droplets just falling on the surface of the object, it can be found that BPs after formic acid treatment are hydrophilic, and the hydrophilicity becomes stronger with additional treatment cycles. Lignin is generally considered to be more hydrophobic than cellulose because it has an aromatic structure and fewer hydroxyl groups. As shown in Figure 1, lignin in BP is removed as the number of treatments increases, and the surface cellulose content increases. The hydroxyl groups in the exposed cellulose interact with water molecules through hydrogen bonds, thereby making the solid surface more hydrophilic [16]. The improvement of hydrophilicity is beneficial to the combination of cellulase and cellulose, which is a likely mechanism for the increased yield of enzymatic hydrolysis noted earlier.

**XRD** Cellulose crystallinity of BPs after formic acid was compared in Figure 3b, and all cellulose in raw and pretreated BPs displayed the typical XRD pattern of cellulose, which exhibited diffraction peaks at approximately 16° and 22.6°. As evidenced by the results indicated that several formic acid pretreatments did not change the cellulose allomorph of BPs. The crystallinity index (CrI) represents the relative content of crystalline cellulose in the total substrate. From Figure 3b, it can be found that after three cycles of formic acid treatment, the CrI value of cellulose in BPs increased from 50.51% to 66.98%. This is because after 4 h formic acid pretreatment, the amorphous region of cellulose and xylan and lignin in the sample are removed, thereby increasing the fraction of crystalline region of cellulose. The continuous treatment of formic acid further began to destroy the crystalline region of cellulose, resulting in a decrease in its CrI value.

It can be speculated from the above results that there may be some stubborn structure bonding together the cellulose, hemicellulose and lignin. Even after multiple treatments with formic acid, hemicellulose and lignin cannot be completely separated by the action of H^+^ and the dissolution ability of formic acid to lignin under the mild condition. Harsher conditions and/or different solvents may be needed to achieve higher hemicellulose and lignin removal. For example, lignin and hemicellulose can be almost completely removed by using strong oxidants or inorganic acid-catalyzed organic reagents to break lignin into small molecules at high temperatures. Zhao et al. [17] used 75 mM sulfuric acid to catalyze γ-valerolactone to completely remove the hemicellulose of Pinus massoniana at 160 °C and only 4.44% of lignin remained and 97.98% lignin was removed.

### 2.4. Characterization of MWL and FAL

**Figure 4:**
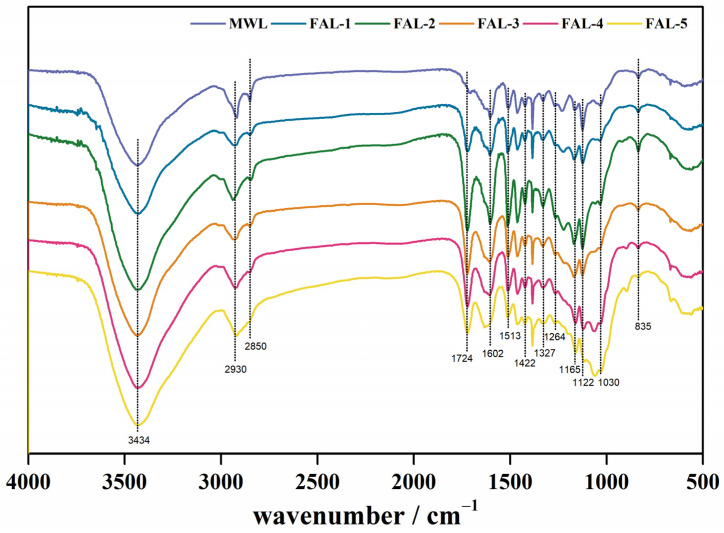
FT-IR of lignin from different pretreated BPs.

**FT-IR** The main functional groups change in MWL and FAL are observed in Figure 4. The broad band near 3434 cm^−1^ is the typical region of O–H stretching in aromatic ring and aliphatic structures (Table 2). The C–H stretching bands at 2930 and 2850 cm^−1^ are attributed to methyl and methylene. The existence of this peak indicates that the extracted lignin has a side chain structure. The–CH_2_ stretching of methylene group at 2850 cm^−1^ gradually disappears with the increase in treatment cycles, which indicates that the dense network structure of lignin in BP is destroyed under the repeated action of formic acid, or the more complex structures are all removed during the earlier cycles of treatment. The C=O valence vibration of adsorbed acetic acid and formic acid is responsible for the visible peaks at 1724 cm^−1^. The peaks at 1602, 1513 and 1422 cm^−1^ were attributed to aromatic skeletal vibrations (C–C) of the lignin, and the peaks of FAL-3, FAL-4 and FAL-5 at 1602 cm^−1^ were weakened, indicating that the structure connected to the benzene ring in the lignin was increasingly destroyed with the increase in treatment cycles. In addition, the S unit with C=O stretching and the G rings with carbonyl stretching appeared at 1327 cm^−1^ and 1264 cm^−1^, respectively, while the bands at 1122 and 1030 cm^−1^ attributed to the aromatic C-H stretching of S and G ring. Aromatic in-plane C–H bending signals can also be found at 1122 cm^−1^ (S) and 1036 cm^−1^ (G), whereas out-of-plane C–H bending of syringyl content is observed at 835 cm^−1^. The band at 1167 cm^−1^ corresponds to C=O in conjugated structure, which is a typical signal of SGH lignin [18]. Compared with MWL, the functional groups of FAL did not change much, which also indicated that the chemical structure of lignin was not modified during the dissolution of lignin by formic acid. Similar phenomena were found in the previous study on the bamboo treated with formic acid [11].

**Figure 5:**
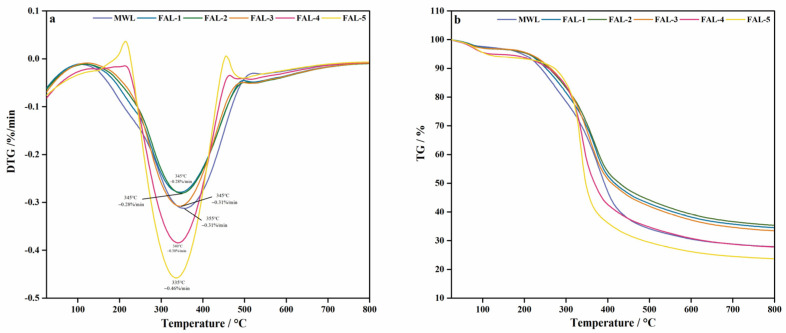
TG and DTG analysis of lignin. (**a**) DTG of BPs, (**b**) TG of BPs.

**TG** The thermogravimetric (TG) and differential thermogravimetric (DTG) curves of the MWL and FALs are shown in Figure 5. According to the literature [18,19,20,21], the decomposition of lignin can be divided into three stages. The first stage is from room temperature to 120 °C, which is due to the dehydration and volatilization of low-molecular weight lignin. The second stage occurs between 200 and 450 °C, during which lignin decomposes rapidly due to the cleavage of inter-unit bonds of lignin and the evaporation of monomeric phenols. At this stage, the weight loss was around 50 wt%. In addition, the maximum weight loss rate of MWL at 355 °C was 0.31%/min, while the maximum weight loss of FAL-1 and FAL-2 occurred at 345 °C, both of which were 0.28%/min. With the increase in treatment cycles, the weight loss rate and weight loss temperature of FAL increased, and the weight loss rate of FAL-5 at 335 °C was 0.46%/min. The results showed that the lignin obtained after BP was treated with formic acid was more difficult to degrade than MWL at first, but with the increase in treatment cycles, the obtained lignin became more easily degraded. The final stage is when the temperature is higher than 450 °C. The weight loss in this stage mainly comes from the further disintegration and recondensation of lignin molecules [22]. At the final temperature of 800 °C, the carbon residue of MWL was 27.9%, while the char residues of FAL-1, FAL-2, and FAL-3 were 34.5%, 35.4% and 33.5%, respectively. FAL-4 and FAL-5 obtained a residual rate of 27.8% and 23.7% (see Table 3). This may be due to the high phenolic hydroxyl group in the lignin obtained by formic acid pretreatment in the previous cycles, resulting in insufficient degradation of lignin [22].

## 3. Materials and Methods

### 3.1. Materials

Bamboo power (BP) used in this study was collected from Anji, Zhejiang and the size was greater than 40 mesh. Formic acid was purchased from Macklin Reagent Co., Ltd. (Shanghai, China), sodium carbonate and 1,4-dioxohexane were purchased from Sinopharm Chemical Reagents Co., Ltd. (Shanghai, China). Cellulase (Cellic^®^ CTec2) was purchased from Sigma (St. Louis, MO, USA).

### 3.2. Formic Acid Pretreatment

**The 1st formic acid pretreatment:** An amount of 160.000 ± 0.005 g of fresh and dried BP and 1600 mL of 90% (*v*/*v*) formic acid were mixed in a glass bottle at a ratio of 1:10, and then the glass bottle was put into a water bath with 120 rpm speed and heated to 85 °C. The bottle was taken out after 4 h of heat preservation and immediately cooled in cold water, followed by solid–liquid separation using a Buchner funnel. The solid residue was first washed with formic acid to remove the surficial sugar and lignin until the filtrate was clear. Then, the solid residue was washed to neutral with distilled water and immersed in 50 g/L sodium carbonate solution for 20 min, and finally washed again to neutral according to previous study [23].

**The 2nd formic acid pretreatment:** An amount of 60.000 ± 0.005 g of the 1st formic acid pretreated BP and 600 mL of 90% formic acid were mixed in a glass bottle at a ratio of 1:10, the reaction condition and the subsequent treatment steps were consistent with the 1st formic acid pretreatment.

**The 3rd–5th formic acid pretreatment:** The pretreated substrates in this experiment were all formic acid pretreated BP obtained in the last formic acid pretreatment, and the reaction condition and the subsequent treatment steps were consistent with the 1st formic acid pretreatment.

### 3.3. Enzymatic Hydrolysis

The pretreated BP obtained from different batches was weighed according to the concentration of 5% substrate and placed in a 50 mL conical flask. Cellulase was added at a dosage of 10 FPU/g glucan, and 1 mL of 1 M sodium citrate buffer was added to control the pH at about 4.8. Finally, the total volume was supplemented with distilled water to 20 mL, and the conical flask was placed in a water bath at 50 °C, 150 rpm for 48 h.

Enzymatic yield (%) = (*C_cellobiose_* × 1.05 + *C_glucose_*) × 100/(*C_glucan_* × 1.1)

where *C_cellobiose_* and *C_glucose_* represent cellobiose and glucose concentrations in the liquid after enzymatic hydrolysis, respectively, 1.05 represents conversion coefficient from cellobiose to glucose, *C_glucan_* represents the intital glucan concentration, 1.1 represents conversion coefficient from glucan to glucose.

### 3.4. Preparation of MWL and FAL

**Milled Wood Lignin (MWL):** Milled Wood Lignin (MWL) of raw BP was extracted using 1,4-dioxane according to previous study [24]. The raw BP was crushed first, and then ball milled with ZrO_2_ at 300 rpm for 12 h. The ball milling conditions were to mill for 5 min and wait for 10 min. The ball-milled raw BP was then weighed in a 500 mL conical flask with 96% (*v*/*v*) 1,4-dioxane at a ratio of 1:10 (g/mL), and the mixture was stirred for 24 h on a magnetic stirrer at room temperature. The raw BP was extracted three times with fresh 1,4-dioxane, and the supernatant was collected by centrifugation (9000 rpm, 10 min). Finally, the mixed extraction was evaporated using a rotary evaporator in a vacuum at 40 °C. The evaporated residue was dissolved in 90% (*v*/*v*) acetic acid and then was dropped into deionized water for precipitation. The MWL was obtained by centrifugation and freeze drying.

**Formic Acid Lignin (FAL):** The formic acid treatment solution obtained from each batch was evaporated using a rotary evaporator in a vacuum at 60 °C to remove formic acid, and then the evaporated residue was dissolved with deionized water. After centrifugation at 8000 rpm for 10 min, the precipitate was collected. Finally, the precipitate was washed three times with deionized water and freeze-dried. FAL obtained from different batch was named FAL-1, FAL-2, FAL-3, FAL-4 and FAL-5.

### 3.5. Characterization of BPs and Lignin

#### 3.5.1. Contact Angle of Raw and Pretreated BPs

The hydrophilicity of raw and pretreated BPs was investigated by water contact angle (WCA) (Dataphysios OCA25, Stuttgart, Germany), which were pre-pressed with an infrared press to produce flakes [25].

#### 3.5.2. XRD of Raw and Pretreated BPs

The crystallinity characteristics of the raw material and pretreated BPs were analyzed by an Ultima IV apparatus (Bruker d8 Advance, Karlsruhe, Germany). The X-ray diffractograms were recorded from diffraction angle 2θ of 5–45° at a scanning speed of 4°/min. Raw and pretreated BPs were crushed and pass through a 100-mesh screen prior to analysis. The crystallinity index (CrI) was calculated using the following formula [26].

CrI = (*I*_002_ − *I_am_*)/*I*_002_ × 100% (1)

where *I*_002_ is the intensity of peak at a 2θ angle close to 22.5° and *I_am_* is the scattering intensity of amorphous fraction at a 2θ angle close to 18°.

#### 3.5.3. FT-IR of Lignin

The structural variations in functional groups in the MWL and FALs were investigated via FT-IR spectrophotometer (NICOLET 8700, Thermo Scientific, Waltham, MA, USA). The FT-IR spectra in the transmittance mode from 4000 to 400 cm^−1^ with a resolution of 4 cm^−1^.

#### 3.5.4. Thermogravimetric Analysis of MWL and FAL

The thermal properties of MWL and different FAL were evaluated by TGA and DTG analysis. To this end, a synchronous thermal analyzer (NETZSCH STA449 F3, Selb, Germany) was used. Heating to 800 °C at a rate of 10 °C/min in a nitrogen atmosphere.

### 3.6. Analysis Methods

#### 3.6.1. Chemical Composition of BP

The components of raw and pretreated BPs were determined by the National Renewable Energy Laboratory standard analytical method [27]. The sample (0.3000 ± 0.0001 g) was dissolved in 3 mL of 72% H_2_SO_4_ at 30 °C and 150 rpm for 1 h. After the low-temperature acidolysis, 84 mL of distilled water was added to the acidolysis bottle to dilute H_2_SO_4_ to 4%. The acidolysis bottle was sealed and placed in an environment of 121 °C for 1 h. After the reaction, the bottle was cooled to room temperature, and the solid–liquid mixture was passed through the G3 sand core funnel to separate solids and liquids. The solids on the sand core funnel were washed to neutral by distilled water and then dried to dryness. The acid-insoluble lignin was calculated by burning the dry solid residue in a muffle furnace at 575 °C. The separated liquid was used to determine glucan, xylan, araban, and acid-insoluble lignin. Hemicellulose was calculated as the sum of xylan and araban.

#### 3.6.2. HPLC Analysis

Concentrations of cellobiose, glucose, xylose and arabinose in the liquid phase were measured by HPLC (Agilent 1260, Santa Clara, CA, USA) equipped with a refractive index detector for calculating enzymatic yield and chemical composition. An aminex HPX-87H (Bio-Rad, Hercules, CA, USA) column was used at 55 °C and 5 mM H_2_SO_4_ was applied as the mobile phase at a flow rate of 0.6 mL/min.

## 4. Conclusions

BP was repeatedly processed with formic acid to better understand the changes in its physical and chemical structure. Even after five cycles of treatments, hemicellulose and lignin were not entirely removed, with residues remaining at 2.5% and 12.1%, respectively. The hydrophobicity of BP rapidly decreased while the cellulose crystallinity increased as treatment cycles rose, and the enzymatic hydrolysis yield rose from 39.6% in the 1st time to 59.3% in the fifth treatment. As treatment cycles increased, FAL decomposed more readily and had lower char residues than MWL.

## Figures and Tables

**Table 1:** Chemical composition changes in pretreated BPs.

	Based on the Raw Material			Based on the Sample in the Last Cycle		
	Cellulose Recovery/%	Hemicellulose Removal/%	Lignin Removal/%	Cellulose Recovery/%	Hemicellulose Removal/%	Lignin Removal/%
1st	94.4 ± 1.8	85.0 ± 0.1	82.5 ± 1.7	94.4 ± 1.8	85.0 ± 0.1	82.5 ± 1.7
2nd	93.0 ± 0.2	91.7 ± 0.1	91.4 ± 0.1	98.6 ± 0.2	44.6 ± 0.4	50.5 ± 0.3
3rd	90.8 ± 0.2	94.2 ± 0.1	93.1 ± 0.1	97.6 ± 0.2	30.6 ± 0.4	19.8 ± 0.9
4th	85.9 ± 0.4	95.4 ± 0.2	93.9 ± 0.1	94.6 ± 0.4	20.3 ± 3.7	12.2 ± 1.2
5th	80.8 ± 0.2	96.3 ± 0.1	94.7 ± 0.1	94.1 ± 0.2	19.2 ± 2.1	12.1 ± 0.4

**Table 2:** Peak assignments for FT-IR spectra.

Wavenumbers/cm^−1^	Assignments
3434	O–H stretch vibration
2930	C–H stretch in –OCH_3_
2850	C–H stretching vibration of –CH_2_
1724	C=O stretch vibration
1602, 1513, 1422	Aromatic skeletal vibrations
1327	S unit
1264	C=O stretch in G units
1165	C=O in conjugated structure in SGH
1122	S units
1030	G units
835	C–H out-of-plane of the S unit and in all positions of H units

**Table 3:** Weight loss and char residues.

Samples	Temperature °C	Weight Loss %/min	Char Residues (800 °C) %
MWL	355	0.31	27.9
FAL-1	345	0.28	34.5
FAL-2	345	0.28	35.4
FAL-3	345	0.31	33.5
FAL-4	340	0.38	27.8
FAL-5	335	0.46	23.7

## Data Availability

Data will be made available on request.
